# PROTOCOL: The effect of education programmes for improving knowledge of back health, ergonomics and postural behaviour in university students: A systematic review

**DOI:** 10.1002/cl2.1213

**Published:** 2022-01-05

**Authors:** Mona Salman, Josette Bettany‐Saltikov, Gokulakannan Kandasamy, Vicki Whittaker, Julie Hogg, Garikoitz Aristegui Racero

**Affiliations:** ^1^ Centre for Public Health, School of Health and Life Sciences (SHLS), Nursing & Midwifery Teesside University Middlesbrough UK; ^2^ Centre for Rehabilitation, School of Health and Life Sciences (SHLS), Allied Health Professions Teesside University Middlesbrough UK; ^3^ Student Life Support (SLS) Teesside University Middlesbrough UK; ^4^ Scoliosis and Posture Centre Donostia San Sebastián Spain

## Abstract

This is the protocol for a Campbell review. The objectives are as follows: To investigate the evidence on the effectiveness of education programmes in improving the knowledge of back health, ergonomics and postural behaviour in University students.

## 1 BACKGROUND

### 1.1 Description of the condition

Musculoskeletal disorders (MSDs) is a term used to describe a group of conditions affecting the bones, muscles and joints (NHS, 2018). According to the Global Burden Disease study in 2016, MSDs were ranked the second main cause of worldwide disability (GBD, 2016). Approximately between 20% and 33% of people all over the world live with a musculoskeletal problem (GBD, 2016). Moreover, the prevalence of MSDs in USA is higher compared to that of cardiovascular diseases representing one in two adults (Bone and Joint initiative USA, 2016). Furthermore, 0.5 million workers suffered from work‐related MSDs in Great Britain in 2017 (Health and Safety Statistics [HSE], 2017).

Several serious physical and mental consequences can result from MSDs. These include lower mobility and ability to perform the daily life activities, reduced social interactions and participation in sport events, more absence from work as well as depression (WHO, 2018). Nevertheless, 10.8 million days of the overall quality of life can be lost because of MSDs (Stringer, 2011). With respect to the healthcare costs, 213 billion dollars representing 1.4% of Gross Domestic Product has been spent in USA in 2011 due to MSDs (Bone and Joint initiative USA, 2016). A similar situation was observed in the UK in the same year where MSDs accounted for more than 25% of all surgeries and 30% of GP visits (Arthritis Research UK, 2013; Department of Health, 2006). This means that they costed almost £4.76 billion of NHS spending each year (Department of Health, 2011).

Although there is a variability in the anthropometric measures between different people, correct posture can be defined as a straight line passing, in the sagittal plane, through the lobe of the ear, the seventh vertebra at the cervical level, the acromion, and the greater trochanter of the femur, anterior to the mid‐line through the knee, and anterior to the lateral malleolus (Kendall et al., 2005). The ideal spinal curvature consists of slight lumbar lordosis and slight thoracic kyphosis (Claus et al., 2009). Several factors such as age, anatomical, pathological, occupational (Johnson, 2012), environmental and psychological factors can influence the ideal posture (Johnson, 2012; Tattersall & Walshaw, 2003). A proper posture is required to prevent strain and stress on the spine as well as to use the body effectively (Claus et al., 2009). Spinal alignment is, therefore, indispensable for healthy skeletal and nervous systems (Szczygieł et al., 2017).

Poor posture is any deviation of the spine from its standard position (Wong & Wong, 2008). Inadequate postural behaviours while carrying loads, put pressure on the muscles and ligaments supporting the spine resulting in increased spinal curvature (NHS, 2018), pain and depression (Canales et al., 2010). Nevertheless, altered head and neck positions may lead to reduced respiratory function (Zafar et al., 2018). For example, patients who live with neck pain have lower respiratory capacity and function (Lane, 2011). This can be explained by the direct effect of excessive forward head posture on the activity of respiratory muscles activities (Okuro et al., 2011). For example, kyphosis leads to compression of the phrenic nerve, supplying the diaphragm, resulting in lower diaphragm activity (Hodges et al., 2002).

Ergonomics is the discipline dealing with well‐designed technology for maximum efficiency and safety (Chang, 2002; Yoser & Mito, 2002) and therefore optimal human well‐being and work performance (Gupta et al., 2014). An appropriate ergonomic design may prevent strain injuries and long‐term disability (Sarkar & Shigli, 2012). Sitting for long periods when using computers leads to kyphosis and forward head inclination (Alattas & Elleithy, 2014) as well as reduced blood flow to bones, muscles, ligaments and tendons resulting in stiffness and pain (Farella et al., 2006).

Health literacy as defined by U.S. Department of Health and Human Services (2000) is ‘the degree to which individuals have the capacity to obtain, process, and understand basic health information and services needed to make appropriate health decisions’. Health awareness increases the individual's self‐care knowledge and skills required for good physical and mental health (DeWalt et al., 2004). Postural kyphosis, for example, can be prevented by learning how to adopt ideal body posture (NHS, 2018). By addressing the postural habits, lower back pain can be reduced (O'Sullivan et al., 2012). Thus, an adequate health knowledge is the most appropriate way to effective self‐management (Briggs & Jordan, 2010).

### 1.2 Description of the intervention

Educational interventions can help people acquire knowledge of the anatomy of the back, how to lift and carry objects effectively, and potential risk factors of low back pain (Sowah et al., 2018). Health promotion is essential in improving disability, pain, as well as the overall quality of life (Albaladejo et al., 2010). Young adults have not been largely targeted by health promotion programmes (Lee et al., 2017). In fact, most of the educational interventions directed for young adults focused on healthy eating and physical activity (Brown et al., 2015; Floegel et al., 2014). Moreover, lack of adulthood health promotion programmes may limit the success of childhood health education interventions (Allman‐Farinelli et al., 2016). To date, there is no consensus on the educational programme for improving knowledge of back health, ergonomics and postural behaviour in University students. Such programmes can vary widely in the literature (Steele et al., 2006). Previous studies in this area may vary in the aims, the ways of delivery, the duration, the intensity and the content of the educational programmes.

#### 1.2.1 Aims of the educational programmes

The objectives of the programmes can include the following: to reduce the prevalence of MSDs (Hoe et al., 2018); to prevent the occurrence of low back pain, to teach acceptable sitting posture, safe lifting techniques, sports injury prevention, to train students to make appropriate and safe decisions regarding the use of their body to prevent the onset of back pain, and to improve their knowledge of back care principles (Steele et al., 2006).

#### 1.2.2 Teachers of back health programmes

The education programmes can be delivered by the following: occupational health and safety professionals (Hoe et al., 2018); physical therapists, physical therapy students, occupational therapists, classroom teachers and physical education teachers (Steele et al., 2006).

#### 1.2.3 Duration and intensity of these programmes

The duration and intensity of the education programmes can range from one 60 min session, one 120 min session, two sessions of 3 h each with a 2‐week interval (Hoe et al., 2018); one 90 min session, one 240 min session, two sessions of 60 min, eight sessions of 120 min, 1 day (Swinton et al., 2017); one 30 min session, six 1 h sessions over 6 weeks, one session with an unspecified duration, 11 sessions over 8 weeks (total 19 h), three sessions of unspecified duration, and 3 years duration with no intensity specified (Steele et al., 2006).

#### 1.2.4 Content of the back‐health programmes

The content of the education programme can include the following: introduction to ergonomics and MSDs, adaptation of the work environment to avoid MSDs, implementation of exercises and relaxation programmes to avoid MSDs, gaining risk assessment skills, information about basic muscle physiology, ideal neutral postures, basic task analysis, recommended office equipment location, recognition of problems related to incorrect equipment placement, adjusting the postural components of the workstation correctly; adjusting the visual components of the workstation correctly; and organising work activities in a preventive manner (Hoe et al., 2018); principles of Swedish back school, anatomy, physiology, spinal care principles and exercises, how to choose, lift and wear backpacks safely, how to recognise when a backpack is too heavy, exercises combined with behavioural intention and self‐monitoring, biomechanics and risk factors for injury as well as how to incorporate this knowledge into everyday life using lifting techniques (Steele et al., 2006).

#### 1.2.5 Ways of delivery of these programmes

The ways of delivery of the programmes can vary widely and include: active learning sessions, discussions and problem‐solving exercises, lectures and informational handouts, demonstrations, simulations (Hoe et al., 2018); web‐based ergonomic education resources (Swinton et al., 2017); lecture on back care (Maher, 2000); incorporated in the curriculum (lectures, worksheets, games, demonstrations, practice, guided self‐discovery and hands‐on activity), posters in the classroom, teacher and parents involvement (Steele et al., 2006).

### 1.3 How the intervention might work

The knowledge of back health, ergonomics and postural behaviour in University students can be underpinned by behaviour change theories as well as learning theories.

Health behaviour model (HBM) suggests that people can change their behaviour positively if they perceive health threat (Rosenstock et al., 1994). HBM is based on perception of five elements: susceptibility, severity, benefits, barriers and self‐efficacy (Glanz et al., 2008). For example, people who attend a back‐care education programme explaining the association between improper postural behaviour and the risk of having musculoskeletal pain may be more able to adapt adequate posture. HBM is one of the most common theories of health behaviour (Orji et al., 2012) and frequently used in health education (Glanz et al., 2008). HBM is widely used in interpreting individuals' attitudes and behaviours in healthcare settings (Ha et al., 2017; Parsa et al., 2017). However, barriers like media, other people's views and the individual's self‐efficacy may influence the effectiveness of this theory (Bandura, 1986). Cognitive behavioural theory (CBT) states that an individual's behaviour is governed by his/her thoughts and feelings (McLeod, 2019). CBT was found to be highly effective in preventing and treating low back pain (Manchikanti, 2010). An randomised control trial (RCT) found that after a 1‐year follow‐up, the cognitive behavioural intervention caused a significant reduction in subacute and chronic low‐back pain as well as in healthcare costs (Lamb et al., 2010). This finding strongly encourages recommending CBT in the low back pain management (Savigny et al., 2009).

Active learning is a way of learning that is centred on the learner not the teacher where the students are engaged in the learning process (Bonwell & Eison, 1991). Critical thinking is not promoted in traditional classes (Schmidt et al., 2015). Furthermore, a major drawback of passive learning is that, according to many educators' observations, students' attention decreases with time during the lecture (Dorestani, 2005). It is recommended that students involved in educational intervention learn by doing and relying on ‘hands‐on’ materials rather than just listening (Hartman et al., 2000).

The literature strongly supports the use of active learning techniques in teaching science (Hsieh, 2013). For example, An RCT of 817 university students investigated the effectiveness of active learning strategies and found improvement in standardised test scores and lower variability in academic performance of active learners compared to traditional learners (Mello & Less, 2013). Therefore, it can be concluded that active learning technique, compared to passive learning, might lead to better results if employed in the educational interventions.

### 1.4 Why it is important to do this review

MSDs represent an important public health problem worldwide and efforts need to be put to tackle this problem. For example, The World Health Organisation has started with the WHO's Global Programme of Work in rehabilitation (WHO, 2018). Moreover, NHS England along with various stakeholders including the Department of Health, the Arthritis and Musculoskeletal Alliance, Arthritis Research UK, and Public Health England, is trying, through a number of interventions, to improve the quality of life of people living with MSDs (NHS, 2018).

To date, there has been little systematic evaluation of the effectiveness of education programmes in improving the knowledge of back health, ergonomics and postural behaviour in University students. A systematic review by Maher (2000) evaluated the effectiveness of workplace interventions in preventing low back pain. Results showed that there is a moderate evidence that education is ineffective in reducing the prevalence and severity of LBP. However, the systematic review was undertaken 21 years ago and did not consider the knowledge of back health and body posture.

A related systematic review by Steele et al. (2006) found that school‐based spinal health interventions might improve spinal care knowledge and reduce prevalence of spinal pain. However, the 12 included papers were of weak quality and the population was different from University students. Furthermore, it was conducted more than 10 years ago.

A recent systematic review by Swinton et al. (2017) which included four RCTs, two non‐randomised, and six pre–post studies, found a limited evidence that ergonomic workplace interventions can improve sitting posture. The Low or very low overall evidence quality of the systematic review needs to be acknowledged. Furthermore, the review was specific to workplace interventions as well as sitting posture.

Hoe et al. (2018) in their very recent systematic review identified 15 RCTs of very low‐ to low‐quality. Results showed that training in ergonomic principles may not prevent work‐related MSDs of the upper limb or neck or both among office workers. It is clear that the review focused on office employees and the upper limb and neck regions.

To the researcher's knowledge, there is no systematic review of the effect of education programmes for improving the knowledge of back health, ergonomics and postural behaviour in University students. Thus, it was thought appropriate to conduct a comprehensive systematic review to help provide policymakers and health practitioners with the most up‐to‐date evidence in this area of research.

## 2 OBJECTIVES

To investigate the evidence on the effectiveness of education programmes in improving the knowledge of back health, ergonomics and postural behaviour in University students.

To explore what set of conditions allow the back‐care educational intervention to be most effective in changing knowledge and behaviour of ergonomics and posture in University students.

## 3 METHODS

### 3.1 Criteria for considering studies for this review

#### 3.1.1 Types of studies

Quantitative studies including RCTs, Cluster randomised controlled trials, quasi‐randomised control trials (QRCTs) and prospective cohort studies with control groups will be included. Single group designs, retrospective case‐control studies and qualitative studies will be excluded. The intervention and control groups can be assigned randomly or nonrandomly. For non‐randomised designs, only a rigorous matched‐comparison group design will be eligible for the analysis whereby the intervention and control groups share similar baseline characteristics (*p* > 0.05 for between group baseline characteristics) and the study is rated as ‘low’ on the ‘adjustment for confounding’ item of the risk of bias assessment. The risk of bias will be assessed using the criteria recommended by the Cochrane Back Review Group (Furlan, Pennick, Bombardier, & van Tulder, 2009; Higgins & Green, 2011), together with items from the Downs and Black (1998) checklist, as outlined in Supporting Information Appendix 5. Control groups can include no educational intervention or educational intervention not related to back care.

#### 3.1.2 Types of participants

For the population, all university students (not college students) with any level of enrolment whether undergraduate or postgraduate students are considered to be eligible to be included. University students with chronic disease or conditions or co morbidities will be excluded.

#### 3.1.3 Types of interventions

Educational interventions on back health, ergonomics and posture will be included. Interventions that include exercises but not education, and educational interventions not related to back care will be excluded. It is any intervention that is universally available to all and university students happen to attend. So, they are not those interventions that are targeted specifically at University students. The requirement for an intervention to be included in this review is that it addresses ergonomics and posture and generates at least one of the study outcomes (knowledge and behaviour of ergonomics and posture).

The contents, duration, intensity and way of delivery of the programme may vary in each of the studies to be included since there is no consensus on the educational programme for improving knowledge of back health, ergonomics and postural behaviour in University students.

#### 3.1.4 Types of outcome measures

To date, there are no standard indicators of outcome measurements to evaluate the effectiveness of educational programmes. Although the aim of the majority of education programmes is a change in a health outcome, that change might occur after the evaluation timeframe. The effectiveness of the intervention will be evaluated at 6 months, 1 and 2 years. We have chosen these follow‐up durations as our review is similar to the review conducted on school children 'School‐based education programmes for improving knowledge of back health, ergonomics and postural behaviour of school children aged 4–18: A systematic review'. By this way, we can compare the findings of both reviews.

##### Primary outcomes

Studies that measure one or more of the following outcomes will be included:

1. Knowledge of ergonomics
2. Knowledge of posture
3. Back care‐related postural behaviours

Any outcomes not listed here and not related to postural behavioural change will be excluded.

##### Secondary outcomes

None.

### 3.2 Search methods for identification of studies

#### 3.2.1 Electronic searches

We will search the following electronic databases to identify research papers.

– The Cumulative Index to Nursing and Allied Health Literature (CINAHL) (via EBSCOhostplatform) (1937 to date)
– MEDLINE (via PubMed) (1809 to present)
– Allied and Complementary MEDicine (AMED) (1983 to 2020)
– SportDiscus (1883 to 2020)
– Embase (via Embase.com) (1996 to 2020)
– Cochrane Central Register of Controlled Trials (CENTRAL) in the Cochrane Library
– Physiotherapy Evidence Database (PEDro)
– The Education Resources Information Centre (ERIC) via EBSCOhost platform (1966 to date)
– Education Source via EBSCOhost platform
– Web of Science (1900 to present)
– SCOPUS

The electronic search strategies for CINAHL, Medline, ERIC and Scopus are outlined in Supporting Information Appendices 1, 2, 3, and 4, respectively. The search will not be restricted to any publication date or language.

#### 3.2.2 Searching other resources

The search across academic databases will be complemented by searching for studies in progress through sources of grey literature (ETHOS, WHO's Library Database (WHOLIS), Web of Science, SCOPUS and ProQuest Dissertations & Theses Global). We will hand search journals (Physiotherapy, Journal of Bodywork and Movement Therapies, Work, European Spine Journal, Spine, Physical Therapy in Sport and International Journal of Musculoskeletal Pain Prevention, Open Journal for Therapy and Rehabilitation, JAMA (The Journal of the American Medical Association), the LANCET and PLOS Medicine) and check the reference lists of included studies to identify additional relevant research papers. We will reach out to authors of included studies as well as the authors identified from the reference lists of the included studies to see if they are familiar with additional studies.

### 3.3 Data collection and analysis

#### 3.3.1 Selection of studies

Using the inclusion and exclusion criteria of this review, a data selection form will be piloted and tested by two independent reviewers for intra‐rater and inter‐rater reliability. These two reviewers will independently screen the titles and abstracts of the search results to select potentially relevant research papers. The eligibility of the obtained full‐text articles will be independently checked by the two review authors who will decide on their inclusion. Discrepancies between the two reviewers will be resolved by the assistance of a third independent reviewer. In case one of the review authors is also the author of a paper, another review author who is not an author of any of the papers will select the studies.

#### 3.3.2 Data extraction and management

Two review authors will independently extract data from the included full‐text articles. In case of inconsistency in the extracted data, the discrepancies will be resolved by a third reviewer. The primary research author will be contacted in the case of missing data for additional information. Studies will be classified as either included, excluded, awaiting assessment, or ongoing.

A standardised data extraction template will be piloted and used to extract the following data from the included studies:

Study design

– Type of study
– Duration of study
– Country of origin

Participants

– Number of participants
– Type of participants
– Level of education

Interventions

– Theory used to explain how the intervention might work: biomedical or biopsychosocial
– Content, Duration, intensity and delivery of the education programme

Outcomes:

Details on the outcome measurements such as definition of the outcome, instruments used to measure the outcome and the period of measurement.

Any of the following outcomes or similar outcomes on knowledge will be extracted:

– Knowledge of spinal curvatures
– Bag characteristics (strap length and limited weight)
– Proper posture when carrying a load
– Proper posture when lifting objects off of the floor
– Proper posture when pushing and pulling objects
– Adequate sleeping posture
– Correct sitting posture
– Best way of sitting while using computers
– Relaxing the back during breaks

Any of the following outcomes or similar outcomes on behaviour will be extracted:

– Bag features (strap length and limited weight)
– Proper posture when carrying a load
– Proper posture when lifting objects off of the floor
– Proper posture when pushing and pulling objects
– Adequate sleeping posture
– Correct sitting posture
– Best way of sitting while using computers
– Relaxing the back during breaks

#### 3.3.3 Assessment of risk of bias in included studies

Two review authors will independently critically appraise the quality of the included full‐text articles. Discrepancies will be resolved by a third reviewer. The risk of bias assessment will be done using the criteria recommended by the Cochrane Back Review Group (Furlan, Pennick, Bombardier, & van Tulder, 2009; Higgins & Green, 2011), together with items from the Downs and Black (1998) checklist, as outlined in Supporting Information Appendix 5. These criteria fall into five bias categories: selection bias, performance bias, attrition bias, detection bias and selective outcome reporting. This risk of bias assessment tool will include the criteria upon which we will decide whether a study will be included or excluded in the review and will also be used to describe the included studies. This criteria refers to the extent to which a study is biased and thus we can make the decision to exclude it from the review. As there are 28 items in the Downs and Black (1998) checklist, studies will be classified into poor (<14), fair (16–19), good (20–25) and excellent (26–28). Studies which are classified as poor and fair will be excluded from the review at the screening stage, whereas studies which are classified as good and excellent will be included in the review. The risk of bias criteria will be scored as high, low or unclear and will be reported in the ‘risk of bias’ table. The overall extent of risk of bias within each bias category will then be rated as ‘Bias’ or ‘No bias’. Whilst it is difficult to provide an exhaustive list of all possible confounding variables at the start of the review, the review authors have experience in this field and are aware of most of the potential confounding variables that may occur when different treatment groups are compared. These may include, for instance, demographic variables such as age, When it comes to grading the quality of the evidence, evidence from studies judged ‘no bias’ for all five categories will not be downgraded. Evidence will be downgraded (−1 point) when three or fewer categories for each study are judged to have bias. Evidence will be downgraded by −2 points when four or more categories for each study are judged to have bias. See Supporting Information Appendices 5 and 7 for the detailed criteria.

#### 3.3.4 Measures of treatment effect

##### Dichotomous data

For dichotomous data, we will calculate odds ratios with a 95% confidence interval (CI) to summarise the findings of each study.

##### Continuous data

For continuous data, we will calculate the mean difference. In case there is heterogeneity in the scales, we will use a standardised mean difference to combine effects across studies (Hedges et al., 2010). When appropriate, we will calculate the missing effect sizes using *p*‐values, standard errors, confidence intervals or *T*‐values through the RevMan calculator (Review Manager (RevMan, 2019). Missing SDs will be computed using data from included studies (Higgins & Green, 2011)

#### 3.3.5 Unit of analysis issues

In case a study consists of more than one intervention group compared to a control group, we will conduct separate meta‐analyses for each intervention. This will be applicable only if enough studies were found. Otherwise, we will combine effect sizes to perform a single pair‐wise comparison (Higgins & Green, 2011). We will use robust variance estimation to adjust for effect size dependency where the authors report data on the same participants across more than one outcome.

#### 3.3.6 Dealing with missing data

In case not all relevant data are reported, missing data will be collected by contacting the authors. If after contacting the authors, data are still found insufficient to be entered into the meta‐analysis, the results will be qualitatively reported under the sections ‘table of characteristics of Included studies’ and ‘summary of findings tables’.

#### 3.3.7 Assessment of heterogeneity

In case the interventions are similar in participants, content of the education programme, intensity and duration of the programme, as well as the outcome measurements, we plan to synthesise the results in a meta‐analysis. We will use a random‐effects model to assess the impact of statistical heterogeneity. We will calculate all overall effects using inverse weighting methods in REVMAN. CMA software will be used to carry out the analysis.

We will assess statistical heterogeneity visually through the forest plots and a standard *χ* ^2^ (*Q*) test. We will also assess statistical heterogeneity using the *τ* ^2^ statistic described in the Cochrane Handbook for Systematic Reviews of Interventions recommendations (Higgins & Green, 2011). These are the criteria to be used: <25% (no heterogeneity); 25%–40%, (low heterogeneity); 50%–74% (moderate heterogeneity); and ≥75% (substantial heterogeneity). We will explore substantial heterogeneity if using subgroup analyses. In addition to the *τ* ^2^ value and the *χ* ^2^ statistic and its p value that consider the direction and magnitude of the effect size, we will also use *τ* ^2^ statistics. We will also include the prediction intervals for the main effects and subgroup analyses using the estimated *τ*.

#### 3.3.8 Assessment of reporting biases

Reporting bias will be assessed and we will use funnel plots in case at least 10 studies are available for the meta‐analysis (Sutton et al., 2000).

#### 3.3.9 Data synthesis

We will report a summary of the included studies and descriptive statistics of the study, participants, interventions and methodological quality characteristics. We will use a random effects model in the analysis through the use of the inverse variance estimation method (Borenstein et al., 2011) as we expect heterogeneity between the studies. We will use RevMan and CMA software to analyse the data collected from the studies. The heterogeneity between subgroups based on age, gender and country of origin will be assessed by performing meta‐regression analyses through CMA software. Published reports that are based on the same study sample will be considered as they may add information to the coding manual. These published reports will be identified as multiple publications when they have same authors, sample sizes, education programmes or outcomes. In this case, we will contact the authors if we are not certain if the original research is a multiple publication to minimise the risk of incorrect weighting of the study.

We will use robust variance estimation method as recommended by Hedges et al. (2010). If the same outcome was measured at different points in time, we will conduct separate meta‐analyses for outcomes measured at different follow‐up periods: before the intervention, weeks after the intervention, and at months, and years of follow‐up. A single summary effect within these time periods will be computed if the outcome measures in the included studies are reported more than once. However, if meta‐analysis is not considered appropriate, we will provide narrative summaries of the studies.

#### 3.3.10 Subgroup analysis and investigation of heterogeneity

We will assess the subgroup analysis by performing the comparison of the following: participant demographics (age 18–23 vs. 24–29, vs. 30–34 or older than 34 years), type of intervention (theoretical or practical) and duration of intervention (hours, days, weeks or months). The subgroup analysis will allow us to generalise the effect of the interventions by age, type of intervention and duration of intervention. The following factors will be explored using subgroup analysis:

1. The type of intervention employed within the study.
2. Follow‐up period: A distinction will be made on the interventions with a shorter follow‐up period (e.g., <4 weeks) to those with a long follow‐up period.

#### 3.3.11 Sensitivity analysis

We will conduct sensitivity analysis to examine if the overall results of data analysis can be influenced by deleting:

Unpublished studies

Studies with outlier effect sizes. Outlier effects will be defined as those ±3 SD beyond the mean. This strategy will allow us to retain most studies, while removing the most extreme effect sizes.

Studies with high risk of bias

Studies with missing information

We will carry out a process sensitivity analysis on domains relating to the quality of the included studies. First, we will exclude quasi‐random studies and include only RCTs. Second, we will remove those studies that need recalculation of the effect size from other statistical tests. Thus, studies with effect sizes calculated directly from means and standard deviations will be left. This may confirm that the way missing data was dealt with was appropriate. Reporting of the process sensitivity analysis will be summarised in a table.

## CONTRIBUTIONS OF AUTHORS

*Substantial contributions to conception and design*: Mona Salman, Josette Bettany‐Saltikov, Gok Kandasamy, Garikotz Aristegui. *Study search and selection*: Julie Hogg, Mona Salman, Josette Bettany‐Saltikov, Gok Kandasamy, Garikotz Aristegui. *Methodological assessment*: Mona Salman, Josette Bettany‐Saltikov, Gok Kandasamy, Garikotz Aristegui. *Acquisition/abstraction of data*: Mona Salman, Josette Bettany‐Saltikov, Gok Kandasamy, Garikotz Aristegui. *Data analysis*: Victoria Whittaker, Mona Salman, Josette Bettany‐Saltikov, Gok Kandasamy, Garikotz Aristegui. *Interpretation of data*: Victoria Whittaker, Mona Salman, Josette Bettany‐Saltikov, Gok Kandasamy, Garikotz Aristegui. *Drafting of the article*: Mona Salman, Josette Bettany‐Saltikov, Gok Kandasamy, Garikotz Aristegui. *Critical revision for important intellectual content*: Mona Salman, Josette Bettany‐Saltikov, Gok Kandasamy, Garikotz Aristegui.

## DECLARATIONS OF INTEREST

All the authors declare that they have no conflict of interest.

## Supporting information

Supporting information.
