# Supplementary material for: PROTOCOL: The effect of education programmes for improving knowledge of back health, ergonomics and postural behaviour in university students: A systematic review
Source: Campbell Syst Rev. 2022 Jan 5;18(1):e1213. doi: 10.1002/cl2.1213 (PMC8732980; doi:10.1002/cl2.1213)
Supplement: Supplementary file 1 — Supporting information. [file CL2-18-e1213-s001.docx]

# Appendices

## 1 Search strategy for CINAHL

S1 University student*

S2 College student*

S3 Undergraduate*

S4 Postgraduate*

S5 Higher education*

S6 Adult*

S7 YoungÃ‚Â adult*

S8 Young people

S9 Emerging adult*

S10= S1 OR S2 OR S3 OR S4 OR S5 OR S6 OR S7 OR S8 OR S9

S11Ã‚Â Health education

S12Ã‚Â safety educationÃ‚Â

S13 health promotion

S14Ã‚Â preventive healthcareÃ‚Â

S15Ã‚Â preventive healthcare services

S16Ã‚Â primary prevention

S17Ã‚Â education

S18Ã‚Â promotion

S19Ã‚Â intervention

S20Ã‚Â initiative

S21Ã‚Â program

S22Ã‚Â presentation

S23Ã‚Â lecture

S24Ã‚Â campaign

S25Ã‚Â instruction

S26=Ã‚Â  S11 OR S12 OR S13 OR S14 OR S15 OR S16 OR S17 OR S18 OR S19 OR S20 OR S21 OR S22 OR S23 OR S24 OR S25

S27Ã‚Â health knowledge

S28 Awareness

S29 Information level*

S30 Health belief*

S31 Safety belief*

S32 Attitude*Ã‚Â

S33Ã‚Â Lifestyle

S34Ã‚Â Life style

S35Ã‚Â Health behaviour*

S36Ã‚Â Risk*

S37Ã‚Â risk taking behaviour*

S38Ã‚Â prevent*

S39Ã‚Â behaviour* change

S40Ã‚Â practice

S41Ã‚Â ergonomics

S42Ã‚Â posture

S43Ã‚Â spin* health

S44Ã‚Â back health

S45Ã‚Â back problem

S46Ã‚Â spin* problem

S47Ã‚Â back care

S48Ã‚Â spin* care

S49Ã‚Â back injur*

S50 spin* injur*

S51back pain

S50 spin* pain

S52Ã‚Â musculoskeletal pain

S53Ã‚Â musculoskeletal injur*

S54= S27 OR S28 OR S29 OR S30 OR S31 OR S32 OR S33 OR S34 OR S35 OR S36 OR S37 OR S38 OR S39 OR S40 OR S41 OR S42 OR S43 OR S44 OR S45 OR S46 OR S47 OR S48 OR S49 OR S50 OR S51 OR S52 OR S53

S55= S10 AND S26 AND S54
